# Supplementary material for: Overexpression and Activation of αvβ3 Integrin Differentially Affects TGFβ2 Signaling in Human Trabecular Meshwork Cells
Source: Cells. 2021 Jul 29;10(8):1923. doi: 10.3390/cells10081923 (PMC8394542; doi:10.3390/cells10081923)
Supplement: Supplementary file 1 [file cells-10-01923-s001.zip › Table S1.pdf]

Table S1. Genes and transcripts uniquely upregulated in cells overexpressing a constitutively active αvβ3 integrin (CAβ3).

Log_2 fold change (FC) values were reported in DGE analysis using EdgeR, and genes and transcripts that demonstrated a $Log_2 \geq 1.5$ FC were identified using R statistical computing and graphics software. Genes and transcripts are listed alphabetically.

Ensembl ID	Gene Symbol	Log_2 FC	Description
**ENSG00000183044**	ABAT	2.256388898	4-aminobutyrate aminotransferase
**ENSG00000085563**	ABCB1	3.532534991	ATP binding cassette subfamily B member 1
**ENSG00000099204**	ABLIM1	1.511933891	actin binding LIM protein 1
**ENSG00000277200**	AC005696.4	2.216741844	novel transcript
**ENSG00000270953**	AC007938.3	2.168835492	novel transcript, antisense to COPG2
**ENSG00000260686**	AC008669.1	1.574254158	novel transcript, overlapping SNX2
**ENSG00000259420**	AC046168.2	1.600563775	novel transcript
**ENSG00000254337**	AC083967.1	3.60611584	novel transcript
**ENSG00000253882**	AC099548.2	1.616126703	family with sequence similarity 115, member C (FAM115C) pseudogene
**ENSG00000280064**	AC130304.1	2.223498783	novel transcript
**ENSG00000280029**	AC244517.11	1.63522868	novel transcript, antisense to multiple protocadherins
**ENSG00000122971**	ACADS	2.286363476	acyl-CoA dehydrogenase short chain
**ENSG00000213088**	ACKR1	1.542919767	atypical chemokine receptor 1 (Duffy blood group)
**ENSG00000150471**	ADGRL3	1.592946906	adhesion G protein-coupled receptor L3
**ENSG00000184160**	ADRA2C	1.734597922	adrenoceptor alpha 2C
**ENSG00000135439**	AGAP2	1.774980569	ArfGAP with GTPase domain, ankyrin repeat and PH domain 2
**ENSG00000244306**	AL589743.1	3.742391421	double homeobox A pseudogene 10
**ENSG00000052850**	ALX4	2.036922117	ALX homeobox 4
**ENSG00000126016**	AMOT	1.866977772	angiomotin
**ENSG00000154188**	ANGPT1	1.755336832	angiopoietin 1
**ENSG00000151150**	ANK3	4.2131673	ankyrin 3
**ENSG00000131620**	ANO1	5.375954302	anoctamin 1
**ENSG00000151572**	ANO4	3.875120707	anoctamin 4
**ENSG00000130208**	APOC1	2.54756142	apolipoprotein C1
**ENSG00000128805**	ARHGAP22	2.242518773	Rho GTPase activating protein 22
**ENSG00000139971**	ARMH4	2.174504872	armadillo-like helical domain containing 4
**ENSG00000140450**	ARRDC4	2.441535194	arrestin domain containing 4
**ENSG00000167654**	ATCAY	2.738693336	ATCAY, caytaxin
**ENSG00000267281**	ATF7	1.502525946	activating transcription factor 7
**ENSG00000125378**	BMP4	1.69373196	bone morphogenetic protein 4

**ENSG00000130303**	BST2	2.323798203	bone marrow stromal cell antigen 2
**ENSG00000204128**	C2orf72	1.762305256	chromosome 2 open reading frame 72
**ENSG00000244731**	C4A	2.256993208	complement C4A (Rodgers blood group)
**ENSG00000233627**	C4A-AS1	2.251498766	C4A antisense RNA 1
**ENSG00000229776**	C4B-AS1	2.251498251	C4B antisense RNA 1
**ENSG00000123838**	C4BPA	2.260810571	complement component 4 binding protein alpha
**ENSG00000167434**	CA4	2.467907325	carbonic anhydrase 4
**ENSG00000007402**	CACNA2D2	3.202452433	calcium voltage-gated channel auxiliary subunit alpha2delta 2
**ENSG00000204682**	CASC10	1.620826016	cancer susceptibility 10
**ENSG00000272168**	CASC15	1.516618832	cancer susceptibility 15
**ENSG00000235505**	CASP17P	1.566517964	caspase 17, pseudogene
**ENSG00000105383**	CD33	4.325270404	CD33 molecule
**ENSG00000113361**	CDH6	1.974390891	cadherin 6
**ENSG00000205403**	CFI	2.224593111	complement factor I
**ENSG00000016391**	CHDH	1.670815676	choline dehydrogenase
**ENSG00000080644**	CHRNA3	1.51109937	cholinergic receptor nicotinic alpha 3 subunit
**ENSG00000160716**	CHRNB2	1.673752734	cholinergic receptor nicotinic beta 2 subunit
**ENSG00000134326**	CMPK2	2.077095115	cytidine/uridine monophosphate kinase 2
**ENSG00000119946**	CNNM1	5.254651159	cyclin and CBS domain divalent metal cation transport mediator 1
**ENSG00000187955**	COL14A1	3.854739343	collagen type XIV alpha 1 chain
**ENSG00000160111**	CPAMD8	2.052001184	C3 and PZP like, alpha-2-macroglobulin domain containing 8
**ENSG00000109846**	CRYAB	1.783410285	crystallin alpha B
**ENSG00000146038**	DCDC2	2.025620559	doublecortin domain containing 2
**ENSG00000166341**	DCHS1	3.649517957	dachsous cadherin-related 1
**ENSG00000203797**	DDO	2.438540365	D-aspartate oxidase
**ENSG00000175984**	DENND2C	1.950651673	DENN domain containing 2C
**ENSG00000162777**	DENND2D	2.633573272	DENN domain containing 2D
**ENSG00000100867**	DHRS2	2.49176263	dehydrogenase/reductase 2
**ENSG00000211448**	DIO2	3.778409274	iodothyronine deiodinase 2
**ENSG00000198719**	DLL1	2.280151082	delta like canonical Notch ligand 1
**ENSG00000108813**	DLX4	1.73845664	distal-less homeobox 4
**ENSG00000161249**	DMKN	2.612747069	dermokine
**ENSG00000176399**	DMRTA1	1.89156894	DMRT like family A1
**ENSG00000108176**	DNAJC12	1.764988821	DnaJ heat shock protein family (Hsp40) member C12
**ENSG00000272636**	DOC2B	1.887570242	double C2 domain beta
**ENSG00000110042**	DTX4	2.00222359	deltex E3 ubiquitin ligase 4
**ENSG00000206195**	DUXAP8	5.462024248	double homeobox A pseudogene 8
**ENSG00000225210**	DUXAP9	4.47599982	double homeobox A pseudogene 9
**ENSG00000164330**	EBF1	1.521780981	EBF transcription factor 1

**ENSG00000088881**	EBF4	1.907401158	EBF family member 4
**ENSG00000115468**	EFHD1	2.412420969	EF-hand domain family member D1
**ENSG00000090776**	EFNB1	1.592375377	ephrin B1
**ENSG00000129521**	EGLN3	2.471333522	egl-9 family hypoxia inducible factor 3
**ENSG00000255150**	EID3	1.503205019	EP300 interacting inhibitor of differentiation 3
**ENSG00000183798**	EMILIN3	2.115591053	elastin microfibril interfacer 3
**ENSG00000149218**	ENDOD1	1.796242981	endonuclease domain containing 1
**ENSG00000110723**	EXPH5	1.581387874	exophilin 5
**ENSG00000148541**	FAM13C	3.445075301	family with sequence similarity 13 member C
**ENSG00000231528**	FAM225A	3.366294664	family with sequence similarity 225 member A
**ENSG00000197872**	FAM49A	5.376146607	family with sequence similarity 49 member A
**ENSG00000168672**	FAM84B	1.684450653	family with sequence similarity 84 member B
**ENSG00000066468**	FGFR2	2.130842703	fibroblast growth factor receptor 2
**ENSG00000068078**	FGFR3	1.579742712	fibroblast growth factor receptor 3
**ENSG00000125848**	FLRT3	2.359363991	fibronectin leucine rich transmembrane protein 3
**ENSG00000179772**	FOXS1	1.522172203	forkhead box S1
**ENSG00000150893**	FREM2	1.619714094	FRAS1 related extracellular matrix protein 2
**ENSG00000175697**	GPR156	1.983585789	G protein-coupled receptor 156
**ENSG00000152402**	GUCY1A2	2.277982645	guanylate cyclase 1 soluble subunit alpha 2
**ENSG00000143512**	HHIPL2	1.575318095	HHIP like 2
**ENSG00000124610**	HIST1H1A	1.713250253	histone cluster 1 H1 family member a
**ENSG00000240929**	HIST2H2BB	2.949068636	histone cluster 2 H2B family member b (pseudogene)
**ENSG00000214922**	HLA-F-AS1	1.98588527	HLA-F antisense RNA 1
**ENSG00000106031**	HOXA13	1.858929908	homeobox A13
**ENSG00000180806**	HOXC9	3.457987431	homeobox C9
**ENSG00000158104**	HPD	2.114728298	4-hydroxyphenylpyruvate dioxygenase
**ENSG00000168453**	HR	1.609404402	HR, lysine demethylase and nuclear receptor corepressor
**ENSG00000185885**	IFITM1	1.5650983	interferon induced transmembrane protein 1
**ENSG00000103742**	IGDCC4	2.565077933	immunoglobulin superfamily DCC subclass member 4
**ENSG00000148798**	INA	1.928314905	internexin neuronal intermediate filament protein alpha
**ENSG00000145703**	IQGAP2	2.585435746	IQ motif containing GTPase activating protein 2
**ENSG00000016082**	ISL1	8.805295588	ISL LIM homeobox 1
**ENSG00000123104**	ITPR2	1.589248734	inositol 1,4,5-trisphosphate receptor type 2
**ENSG00000167216**	KATNAL2	2.632260749	katanin catalytic subunit A1 like 2
**ENSG00000107821**	KAZALD1	1.606322463	Kazal type serine peptidase inhibitor domain 1
**ENSG00000176595**	KBTBD11	1.714191629	kelch repeat and BTB domain containing 11

**ENSG00000121361**	KCNJ8	1.861351116	potassium voltage-gated channel subfamily J member 8
**ENSG00000162687**	KCNT2	1.891171698	potassium sodium-activated channel subfamily T member 2
**ENSG00000171435**	KSR2	2.088628339	kinase suppressor of ras 2
**ENSG00000115919**	KYNU	2.626773304	kynureninase
**ENSG00000125869**	LAMP5	8.00250157	lysosomal associated membrane protein family member 5
**ENSG00000273706**	LHX1	3.069492146	LIM homeobox 1
**ENSG00000106689**	LHX2	2.021636565	LIM homeobox 2
**ENSG00000111052**	LIN7A	1.812356832	lin-7 homolog A, crumbs cell polarity complex component
**ENSG00000223764**	LINC02593	2.198344283	long intergenic non-protein coding RNA 2593
**ENSG00000165379**	LRFN5	1.620322171	leucine rich repeat and fibronectin type III domain containing 5
**ENSG00000150556**	LYPD6B	4.182986106	LY6/PLAUR domain containing 6B
**ENSG00000187601**	MAGEH1	1.964737303	MAGE family member H1
**ENSG00000152939**	MARVELD2	2.541414652	MARVEL domain containing 2
**ENSG00000106484**	MEST	2.178071159	mesoderm specific transcript
**ENSG00000166482**	MFAP4	2.632730155	microfibril associated protein 4
**ENSG00000116990**	MYCL	1.880524154	MYCL proto-oncogene, bHLH transcription factor
**ENSG00000141052**	MYOCD	2.935712892	myocardin
**ENSG00000154654**	NCAM2	1.778094671	neural cell adhesion molecule 2
**ENSG00000064300**	NGFR	1.517925194	nerve growth factor receptor
**ENSG00000084628**	NKAIN1	2.702347811	sodium/potassium transporting ATPase interacting 1
**ENSG00000104967**	NOVA2	2.287692452	NOVA alternative splicing regulator 2
**ENSG00000113389**	NPR3	1.703228397	natriuretic peptide receptor 3
**ENSG00000196368**	NUDT11	3.42875713	nudix hydrolase 11
**ENSG00000089127**	OAS1	2.036647565	2'-5'-oligoadenylate synthetase 1
**ENSG00000111335**	OAS2	3.445520531	2'-5'-oligoadenylate synthetase 2
**ENSG00000130558**	OLFM1	2.543914395	olfactomedin 1
**ENSG00000089041**	P2RX7	2.132740668	purinergic receptor P2X 7
**ENSG00000007372**	PAX6	2.78355498	paired box 6
**ENSG00000156453**	PCDH1	2.474464969	protocadherin 1
**ENSG00000189184**	PCDH18	2.023322132	protocadherin 18
**ENSG00000197479**	PCDHB11	2.088211962	protocadherin beta 11
**ENSG00000120327**	PCDHB14	1.856173495	protocadherin beta 14
**ENSG00000172572**	PDE3A	2.813567906	phosphodiesterase 3A
**ENSG00000138735**	PDE5A	1.673816237	phosphodiesterase 5A
**ENSG00000113231**	PDE8B	1.605860331	phosphodiesterase 8B
**ENSG00000135549**	PKIB	1.8537746	cAMP-dependent protein kinase inhibitor beta

**ENSG00000268894**	PLCE1-AS1	1.735792919	PLCE1 antisense RNA 1
**ENSG00000109819**	PPARGC1A	1.634813801	PPARG coactivator 1 alpha
**ENSG00000156475**	PPP2R2B	1.611738903	protein phosphatase 2 regulatory subunit Bbeta
**ENSG00000164099**	PRSS12	1.734692849	serine protease 12
**ENSG00000184489**	PTP4A3	1.691988544	protein tyrosine phosphatase type IVA, member 3
**ENSG00000122035**	RASL11A	1.642121215	RAS like family 11 member A
**ENSG00000068615**	REEP1	1.57995915	receptor accessory protein 1
**ENSG00000178882**	RFLNA	1.618194549	refilin A
**ENSG00000182175**	RGMA	2.162076007	repulsive guidance molecule BMP co-receptor a
**ENSG00000135828**	RNASEL	2.141930762	ribonuclease L
**ENSG00000108309**	RUNDC3A	1.676870543	RUN domain containing 3A
**ENSG00000180739**	S1PR5	1.904371805	sphingosine-1-phosphate receptor 5
**ENSG00000187634**	SAMD11	1.851289718	sterile alpha motif domain containing 11
**ENSG00000203727**	SAMD5	1.648288014	sterile alpha motif domain containing 5
**ENSG00000169432**	SCN9A	1.647541827	sodium voltage-gated channel alpha subunit 9
**ENSG00000250722**	SELENOP	3.82421175	selenoprotein P
**ENSG00000153993**	SEMA3D	1.502617479	semaphorin 3D
**ENSG00000092421**	SEMA6A	1.552206964	semaphorin 6A
**ENSG00000137872**	SEMA6D	4.348299959	semaphorin 6D
**ENSG00000135917**	SLC19A3	1.5873819	solute carrier family 19 member 3
**ENSG00000198246**	SLC29A3	1.985607806	solute carrier family 29 member 3
**ENSG00000013293**	SLC7A14	3.110401996	solute carrier family 7 member 14
**ENSG00000118160**	SLC8A2	2.207330186	solute carrier family 8 member A2
**ENSG00000137571**	SLCO5A1	2.570592907	solute carrier organic anion transporter family member 5A1
**ENSG00000184347**	SLIT3	1.580583262	slit guidance ligand 3
**ENSG00000184564**	SLITRK6	2.292534183	SLIT and NTRK like family member 6
**ENSG00000137834**	SMAD6	2.194589458	SMAD family member 6
**ENSG00000064692**	SNCAIP	2.36271367	synuclein alpha interacting protein
**ENSG00000162804**	SNED1	1.585079371	sushi, nidogen and EGF like domains 1
**ENSG00000095637**	SORBS1	2.150794711	sorbin and SH3 domain containing 1
**ENSG00000134532**	SOX5	3.48665113	SRY-box 5
**ENSG00000152377**	SPOCK1	1.929077759	SPARC (osteonectin), cwcv and kazal like domains proteoglycan 1
**ENSG00000196220**	SRGAP3	2.812733165	SLIT-ROBO Rho GTPase activating protein 3
**ENSG00000263465**	SRSF8	1.811135417	serine and arginine rich splicing factor 8
**ENSG00000180616**	SSTR2	2.280986901	somatostatin receptor 2
**ENSG00000126561**	STAT5A	3.324090059	signal transducer and activator of transcription 5A
**ENSG00000137573**	SULF1	1.75927283	sulfatase 1

**ENSG00000161860**	SYCE2	2.186703216	synaptonemal complex central element protein 2
**ENSG00000067715**	SYT1	2.003864654	synaptotagmin 1
**ENSG00000019505**	SYT13	6.697589635	synaptotagmin 13
**ENSG00000092969**	TGFB2	1.626821497	transforming growth factor beta 2
**ENSG00000038295**	TLL1	4.192038345	tolloid like 1
**ENSG00000136404**	TM6SF1	1.630764059	transmembrane 6 superfamily member 1
**ENSG00000166448**	TMEM130	1.906319573	transmembrane protein 130
**ENSG00000165548**	TMEM63C	2.830172274	transmembrane protein 63C
**ENSG00000158164**	TMSB15A	2.838873916	thymosin beta 15a
**ENSG00000198846**	TOX	1.719319293	thymocyte selection associated high mobility group box
**ENSG00000009790**	TRAF3IP3	2.134856223	TRAF3 interacting protein 3
**ENSG00000067445**	TRO	1.525528714	trophinin
**ENSG00000110900**	TSPAN11	2.630188384	tetraspanin 11
**ENSG00000106537**	TSPAN13	1.566201983	tetraspanin 13
**ENSG00000180543**	TSPYL5	4.230885119	TSPY like 5
**ENSG00000265972**	TXNIP	3.636536214	thioredoxin interacting protein
**ENSG00000182040**	USH1G	2.221624322	USH1 protein network component sans
**ENSG00000247746**	USP51	4.022077134	ubiquitin specific peptidase 51
**ENSG00000110002**	VWA5A	2.672650886	von Willebrand factor A domain containing 5A
**ENSG00000075290**	WNT8B	1.904178673	Wnt family member 8B
**ENSG00000139800**	ZIC5	1.790234979	Zic family member 5
**ENSG00000197937**	ZNF347	1.778591999	zinc finger protein 347
**ENSG00000124613**	ZNF391	1.928408869	zinc finger protein 391
**ENSG00000197013**	ZNF429	1.55858418	zinc finger protein 429
**ENSG00000181444**	ZNF467	2.758067157	zinc finger protein 467
**ENSG00000168916**	ZNF608	2.634700126	zinc finger protein 608
**ENSG00000167528**	ZNF641	2.166121039	zinc finger protein 641
**ENSG00000160229**	ZNF66	1.669039302	zinc finger protein 66
**ENSG00000198046**	ZNF667	1.685608926	zinc finger protein 667
**ENSG00000183850**	ZNF730	1.556097598	zinc finger protein 730
