# Supplementary material for: Overexpression and Activation of αvβ3 Integrin Differentially Affects TGFβ2 Signaling in Human Trabecular Meshwork Cells
Source: Cells. 2021 Jul 29;10(8):1923. doi: 10.3390/cells10081923 (PMC8394542; doi:10.3390/cells10081923)
Supplement: Supplementary file 1 [file cells-10-01923-s001.zip › Table S2.pdf]

Table S2. Genes and transcripts uniquely downregulated in cells overexpressing a constitutively active αvβ3 integrin (CAβ3).

Log_2 fold change (FC) values were reported in DGE analysis using EdgeR, and genes and transcripts that demonstrated a $Log_2 \leq -1.5$ FC were identified using R statistical computing and graphics software. Genes and transcripts are listed alphabetically.

Ensembl ID	Gene Symbol	Log_2 FC	Description
**ENSG00000268812**	AC004264.1	-1.991396937	novel transcript, antisense to LIF
**ENSG00000254887**	AC010247.1	-1.820701391	novel transcript, antisense to POU2F2
**ENSG00000256861**	AC048338.1	-8.836046928	novel transcript
**ENSG00000258232**	AC125611.3	-10.75481719	novel transcript, antisense to TUBA1C
**ENSG00000215861**	AC245297.1	-1.828444904	phosphodiesterase 4D interacting protein (myomegalin) (PDE4DIP) pseudogene
**ENSG00000224945**	AL353150.1	-2.211061382	novel transcript
**ENSG00000154065**	ANKRD29	-1.806241919	ankyrin repeat domain 29
**ENSG00000138772**	ANXA3	-3.09451016	annexin A3
**ENSG00000138639**	ARHGAP24	-2.039246148	Rho GTPase activating protein 24
**ENSG00000184867**	ARMCX2	-2.010051376	armadillo repeat containing X-linked 2
**ENSG00000023445**	BIRC3	-1.751782753	baculoviral IAP repeat containing 3
**ENSG00000164619**	BMPER	-2.067122128	BMP binding endothelial regulator
**ENSG00000169594**	BNC1	-1.8888467	basonuclin 1
**ENSG00000120217**	CD274	-1.829161111	CD274 molecule
**ENSG00000163814**	CDCP1	-3.72977362	CUB domain containing protein 1
**ENSG00000183196**	CHST6	-1.692985225	carbohydrate sulfotransferase 6
**ENSG00000147119**	CHST7	-1.814708957	carbohydrate sulfotransferase 7
**ENSG00000113805**	CNTN3	-3.173118934	contactin 3
**ENSG00000197467**	COL13A1	-1.64042802	collagen type XIII alpha 1 chain
**ENSG00000108342**	CSF3	-3.690256759	colony stimulating factor 3
**ENSG00000163739**	CXCL1	-1.991292869	C-X-C motif chemokine ligand 1
**ENSG00000081041**	CXCL2	-2.213041858	C-X-C motif chemokine ligand 2
**ENSG00000163734**	CXCL3	-3.003403221	C-X-C motif chemokine ligand 3
**ENSG00000163735**	CXCL5	-1.860018019	C-X-C motif chemokine ligand 5
**ENSG00000169429**	CXCL8	-2.853844759	C-X-C motif chemokine ligand 8
**ENSG00000107984**	DKK1	-1.914148292	dickkopf WNT signaling pathway inhibitor 1
**ENSG00000128590**	DNAJB9	-1.558079736	DnaJ heat shock protein family (Hsp40) member B9
**ENSG00000187957**	DNER	-1.781751986	delta/notch like EGF repeat containing
**ENSG00000134769**	DTNA	-2.34651843	dystrobrevin alpha
**ENSG00000138166**	DUSP5	-1.573654655	dual specificity phosphatase 5
**ENSG00000134531**	EMP1	-1.875691721	epithelial membrane protein 1
**ENSG00000185862**	EVI2B	-1.98384939	ecotropic viral integration site 2B

**ENSG00000064655**	EYA2	-2.063825752	EYA transcriptional coactivator and phosphatase 2
**ENSG00000103089**	FA2H	-1.516395866	fatty acid 2-hydroxylase
**ENSG00000154319**	FAM167A	-2.22351973	family with sequence similarity 167 member A
**ENSG00000162746**	FCRLB	-1.582633139	Fc receptor like B
**ENSG00000175592**	FOSL1	-1.983301242	FOS like 1, AP-1 transcription factor subunit
**ENSG00000174500**	GCSAM	-1.564754727	germinal center associated signaling and motility
**ENSG00000166923**	GREM1	-1.965465728	gremlin 1, DAN family BMP antagonist
**ENSG00000189369**	GSPT2	-2.811460806	G1 to S phase transition 2
**ENSG00000274290**	HIST1H2BE	-2.511407222	histone cluster 1 H2B family member e
**ENSG00000115756**	HPCAL1	-1.543022612	hippocalcin like 1
**ENSG00000148680**	HTR7	-1.585619731	5-hydroxytryptamine receptor 7
**ENSG00000095752**	IL11	-2.047710214	interleukin 11
**ENSG00000125538**	IL1B	-3.954943908	interleukin 1 beta
**ENSG00000164509**	IL31RA	-3.457925362	interleukin 31 receptor A
**ENSG00000163083**	INHBB	-1.648769502	inhibin subunit beta B
**ENSG00000151364**	KCTD14	-2.789059168	potassium channel tetramerization domain containing 14
**ENSG00000178202**	KDELC2	-1.718428643	KDEL motif containing 2
**ENSG00000127528**	KLF2	-1.538195537	Kruppel like factor 2
**ENSG00000205809**	KLRC2	-1.678061452	killer cell lectin like receptor C2
**ENSG00000128342**	LIF	-2.572899451	LIF, interleukin 6 family cytokine
**ENSG00000246430**	LINC00968	-1.790599633	long intergenic non-protein coding RNA 968
**ENSG00000242147**	LINC02657	-1.675213447	long intergenic non-protein coding RNA 2657
**ENSG00000110031**	LPXN	-1.507356275	leupaxin
**ENSG00000153714**	LURAP1L	-2.424505767	leucine rich adaptor protein 1 like
**ENSG00000185022**	MAFF	-1.841816751	MAF bZIP transcription factor F
**ENSG00000197971**	MBP	-2.622446775	myelin basic protein
**ENSG00000168389**	MFSD2A	-1.599721227	major facilitator superfamily domain containing 2A
**ENSG00000253522**	MIR3142HG	-1.594581074	MIR3142 host gene
**ENSG00000196549**	MME	-1.503242387	membrane metalloendopeptidase
**ENSG00000102854**	MSLN	-1.602678703	mesothelin
**ENSG00000133055**	MYBPH	-1.519648684	myosin binding protein H
**ENSG00000229644**	NAMPTP1	-1.545856696	nicotinamide phosphoribosyltransferase pseudogene 1
**ENSG00000122585**	NPY	-2.570075417	neuropeptide Y
**ENSG00000118257**	NRP2	-2.271274726	neuropilin 2
**ENSG00000177144**	NUDT4B	-1.529161539	nudix hydrolase 4B
**ENSG00000226328**	NUP50-DT	-2.144345686	NUP50 divergent transcript

**ENSG00000137819**	PAQR5	-1.861771537	progestin and adipoQ receptor family member 5
**ENSG00000188677**	PARVB	-2.553371268	parvin beta
**ENSG00000253731**	PCDHGA6	-1.602625701	protocadherin gamma subfamily A, 6
**ENSG00000175426**	PCSK1	-1.725823785	proprotein convertase subtilisin/kexin type 1
**ENSG00000258429**	PDF	-1.594087122	peptide deformylase, mitochondrial
**ENSG00000177614**	PGBD5	-2.035902229	piggyBac transposable element derived 5
**ENSG00000181649**	PHLDA2	-1.897784122	pleckstrin homology like domain family A member 2
**ENSG00000100558**	PLEK2	-2.061318736	pleckstrin 2
**ENSG00000119698**	PPP4R4	-1.786939032	protein phosphatase 4 regulatory subunit 4
**ENSG00000106772**	PRUNE2	-2.019538158	prune homolog 2 with BCH domain
**ENSG00000148344**	PTGES	-2.23429819	prostaglandin E synthase
**ENSG00000151490**	PTPRO	-1.79152749	protein tyrosine phosphatase, receptor type O
**ENSG00000041353**	RAB27B	-1.567298236	RAB27B, member RAS oncogene family
**ENSG00000104856**	RELB	-1.508096473	RELB proto-oncogene, NF-kB subunit
**ENSG00000277739**	RF00002	-1.660318574	NA
**ENSG00000196460**	RFX8	-3.976835018	RFX family member 8, lacking RFX DNA binding domain
**ENSG00000173156**	RHOD	-1.734142397	ras homolog family member D
**ENSG00000278771**	RN7SL3	-2.010134682	RNA, 7SL, cytoplasmic 3
**ENSG00000278189**	RNA5-8SN1	-1.660318578	RNA, 5.8S ribosomal N1
**ENSG00000278233**	RNA5-8SN2	-1.660318581	RNA, 5.8S ribosomal N2
**ENSG00000275215**	RNA5-8SN3	-1.660318568	RNA, 5.8S ribosomal N3
**ENSG00000206585**	RNVU1-7	-1.823893721	RNA, variant U1 small nuclear 7
**ENSG00000197956**	S100A6	-1.618913379	S100 calcium binding protein A6
**ENSG00000189001**	SBSN	-1.870212129	suprabasin
**ENSG00000171951**	SCG2	-2.102123269	secretogranin II
**ENSG00000106366**	SERPINE1	-1.705199473	serpin family E member 1
**ENSG00000118473**	SGIP1	-1.991229357	SH3 domain GRB2 like endophilin interacting protein 1
**ENSG00000189410**	SH2D5	-1.557658489	SH2 domain containing 5
**ENSG00000259863**	SH3RF3-AS1	-1.614951605	SH3RF3 antisense RNA 1
**ESG00000142178**	SIK1	-1.501009133	salt inducible kinase 1
**ENSG00000265185**	SNORD3B-1	-1.538402261	small nucleolar RNA, C/D box 3B-1
**ENSG00000262074**	SNORD3B-2	-1.6161787	small nucleolar RNA, C/D box 3B-2
**ENSG00000079263**	SP140	-1.877421242	SP140 nuclear body protein
**ENSG00000134668**	SPOCD1	-2.582502785	SPOC domain containing 1
**ENSG00000117069**	ST6GALNAC5	-1.736569499	ST6 N-acetylgalactosaminide alpha-2,6-sialyltransferase 5
**ENSG00000164674**	SYTL3	-1.604198478	synaptotagmin like 3
**ENSG00000158246**	TENT5B	-2.138860226	terminal nucleotidyltransferase 5B

**ENSG00000133863**	TEX15	-2.577584943	testis expressed 15, meiosis and synapsis associated
**ENSG00000145107**	TM4SF19	-2.005702078	transmembrane 4 L six family member 19
**ENSG00000249992**	TMEM158	-2.126264863	transmembrane protein 158 (gene/pseudogene)
**ENSG00000118503**	TNFAIP3	-1.917747885	TNF alpha induced protein 3
**ENSG00000123610**	TNFAIP6	-2.804564769	TNF alpha induced protein 6
**ENSG00000179046**	TRIML2	-1.952947411	tripartite motif family like 2
**ENSG00000162692**	VCAM1	-2.070676141	vascular cell adhesion molecule 1
**ENSG00000112715**	VEGFA	-2.110359862	vascular endothelial growth factor A
**ENSG00000136059**	VILL	-3.303321858	villin like
**ENSG00000188064**	WNT7B	-1.820245207	Wnt family member 7B
