# Supplementary material for: Overexpression and Activation of αvβ3 Integrin Differentially Affects TGFβ2 Signaling in Human Trabecular Meshwork Cells
Source: Cells. 2021 Jul 29;10(8):1923. doi: 10.3390/cells10081923 (PMC8394542; doi:10.3390/cells10081923)
Supplement: Supplementary file 1 [file cells-10-01923-s001.zip › Table S3.pdf]

Table S3. Genes and transcripts uniquely upregulated in cells overexpressing a wild type αvβ3 integrin (WTβ3).

Log_2 fold change (FC) values were reported in DGE analysis using EdgeR, and genes and transcripts that demonstrated a $Log_2 \geq 1.5$ FC were identified using R statistical computing and graphics software. Genes and transcripts are listed alphabetically.

Ensembl ID	Gene Symbol	Log_2 FC	Description
**ENSG00000277013**	AC008556.1	1.657490119	novel transcript
**ENSG00000184060**	ADAP2	3.073455205	ArfGAP with dual PH domains 2
**ENSG00000181790**	ADGRB1	3.106749323	adhesion G protein-coupled receptor B1
**ENSG00000270149**	AL591806.3	8.436492105	novel transcript, TSTD1 - F11R readthrough
**ENSG00000132932**	ATP8A2	2.164585395	ATPase phospholipid transporting 8A2
**ENSG00000169255**	B3GALNT1	2.164585395	beta-1,3-N-acetylgalactosaminyltransferase 1 (globoside blood group)
**ENSG00000107159**	CA9	2.433175167	carbonic anhydrase 9
**ENSG00000198286**	CARD11	2.690665088	caspase recruitment domain family member 11
**ENSG00000163814**	CDCP1	3.17818399	CUB domain containing protein 1
**ENSG00000087237**	CETP	2.085407427	cholesteryl ester transfer protein
**ENSG00000171812**	COL8A2	2.714186924	collagen type VIII alpha 2 chain
**ENSG00000021826**	CPS1	1.564300489	carbamoyl-phosphate synthase 1
**ENSG00000231890**	DARS-AS1	1.552175281	DARS antisense RNA 1
**ENSG00000162595**	DIRAS3	1.935173921	DIRAS family GTPase 3
**ENSG00000138080**	EMILIN1	1.508876998	elastin microfibril interfacer 1
**ENSG00000001561**	ENPP4	5.009830622	ectonucleotide pyrophosphatase/phosphodiesterase 4
**ENSG00000088340**	FER1L4	1.796113673	fer-1 like family member 4, pseudogene
**ENSG00000280614**	FP236383.2	4.542337024	novel transcript, similar to YY1 associated myogenesis RNA 1 YAM1
**ENSG00000281181**	FP236383.3	4.542337024	novel transcript, similar to YY1 associated myogenesis RNA 1 YAM1
**ENSG00000280800**	FP671120.4	4.542337024	novel transcript, similar to YY1 associated myogenesis RNA 1 YAM1
**ENSG00000188158**	NHS	1.540047318	NHS actin remodeling regulator
**ENSG00000109255**	NMU	1.704840634	neuromedin U
**ENSG00000254221**	PCDHGB1	1.589513607	protocadherin gamma subfamily B, 1
**ENSG00000262209**	PCDHGB3	1.728711002	protocadherin gamma subfamily B, 3
**ENSG00000145287**	PLAC8	1.531485644	placenta specific 8
**ENSG00000123560**	PLP1	1.51588585	proteolipid protein 1
**ENSG00000133110**	POSTN	3.883827359	periostin
**ENSG00000124212**	PTGIS	1.885897532	prostaglandin I2 synthase
**ENSG00000115828**	QPCT	1.697237523	glutaminyl-peptide cyclotransferase

**ENSG00000162444**	RBP7	1.925243446	retinol binding protein 7
**ENSG00000277739**	RF00002	3.077239388	NA
**ENSG00000278189**	RNA5-8SN1	3.077239388	RNA, 5.8S ribosomal N1
**ENSG00000278233**	RNA5-8SN2	3.077239388	RNA, 5.8S ribosomal N2
**ENSG00000275215**	RNA5-8SN3	3.077239388	RNA, 5.8S ribosomal N3
**ENSG00000108830**	RND2	2.915506786	Rho family GTPase 2
**ENSG00000234498**	RPL13AP20	1.827936454	ribosomal protein L13a pseudogene 20
**ENSG00000082684**	SEMA5B	1.767279762	semaphorin 5B
**ENSG00000170542**	SERPINB9	3.241317149	serpin family B member 9
**ENSG00000107295**	SH3GL2	2.298341516	SH3 domain containing GRB2 like 2, endophilin A1
**ENSG00000237914**	SIRPG-AS1	2.12244344	SIRPG antisense RNA 1
**ENSG00000204385**	SLC44A4	2.745910205	solute carrier family 44 member 4
**ENSG00000092068**	SLC7A8	1.93808603	solute carrier family 7 member 8
**ENSG00000061656**	SPAG4	1.742403165	sperm associated antigen 4
**ENSG00000133138**	TBC1D8B	1.621959679	TBC1 domain family member 8B
**ENSG00000165929**	TC2N	1.509119834	tandem C2 domains, nuclear
**ENSG00000149256**	TENM4	4.540006466	teneurin transmembrane protein 4
**ENSG00000127824**	TUBA4A	1.664284445	tubulin alpha 4a
**ENSG00000188171**	ZNF626	1.929448374	zinc finger protein 626
