# Supplementary material for: Overexpression and Activation of αvβ3 Integrin Differentially Affects TGFβ2 Signaling in Human Trabecular Meshwork Cells
Source: Cells. 2021 Jul 29;10(8):1923. doi: 10.3390/cells10081923 (PMC8394542; doi:10.3390/cells10081923)
Supplement: Supplementary file 1 [file cells-10-01923-s001.zip › Table S4.pdf]

Table S4. Genes and transcripts uniquely downregulated in cells overexpressing a wild type αvβ3 integrin (WTβ3).

Log_2 fold change (FC) values were reported in DGE analysis using EdgeR, and genes and transcripts that demonstrated a $Log_2 \leq -1.5$ FC were identified using R statistical computing and graphics software. Genes and transcripts are listed alphabetically.

Ensembl ID	Gene Symbol	Log_2 FC	Description
**ENSG00000261114**	AC012181.1	-1.648797136	novel transcript, sense intronic to HERPUD1
**ENSG00000226084**	AC113935.1	-1.879357636	ribosomal protein L17 (RPL17) pseudogene
**ENSG00000163017**	ACTG2	-2.039849424	actin, gamma 2, smooth muscle, enteric
**ENSG00000187068**	C3orf70	-2.599340256	chromosome 3 open reading frame 70
**ENSG00000140937**	CDH11	-3.118218115	cadherin 11
**ENSG00000250479**	CHCHD10	-2.164889951	coiled-coil-helix-coiled-coil-helix domain containing 10
**ENSG00000060718**	COL11A1	-1.95561168	collagen type XI alpha 1 chain
**ENSG00000112297**	CRYBG1	-1.554796347	crystallin beta-gamma domain containing 1
**ENSG00000138061**	CYP1B1	-2.283155586	cytochrome P450 family 1 subfamily B member 1
**ENSG00000232973**	CYP1B1-AS1	-1.709108114	CYP1B1 antisense RNA 1
**ENSG00000164330**	EBF1	-2.330896568	EBF transcription factor 1
**ENSG00000122877**	EGR2	-1.524158214	early growth response 2
**ENSG00000158089**	GALNT14	-1.542472136	polypeptide N-acetylgalactosaminyltransferase 14
**ENSG00000168621**	GDNF	-2.191316189	glial cell derived neurotrophic factor
**ENSG00000183671**	GPR1	-1.732885264	G protein-coupled receptor 1
**ENSG00000138646**	HERC5	-3.863132591	HECT and RLD domain containing E3 ubiquitin protein ligase 5
**ENSG00000103522**	IL21R	-1.813038218	interleukin 21 receptor
**ENSG00000154721**	JAM2	-1.543157057	junctional adhesion molecule 2
**ENSG00000170745**	KCNS3	-1.543157057	potassium voltage-gated channel modifier subfamily S member 3
**ENSG00000196872**	KIAA1211L	-1.97123368	KIAA1211 like
**ENSG00000128606**	LRRC17	-3.979037697	leucine rich repeat containing 17
**ENSG00000135525**	MAP7	-2.067185538	microtubule associated protein 7
**ENSG00000165175**	MID1IP1	-1.723597971	MID1 interacting protein 1
**ENSG00000184117**	NIPSNAP1	-1.513382462	nipsnap homolog 1
**ENSG00000176046**	NUPR1	-2.466179682	nuclear protein 1, transcriptional regulator
**ENSG00000116329**	OPRD1	-2.301979203	opioid receptor delta 1
**ENSG00000280165**	PCDH20	-1.627909227	protocadherin 20
**ENSG00000196155**	PLEKHG4	-2.538901757	pleckstrin homology and RhoGEF domain containing G4

**ENSG00000054356**	PTPRN	-2.208669559	protein tyrosine phosphatase, receptor type N
**ENSG00000100228**	RAB36	-2.46722703	RAB36, member RAS oncogene family
**ENSG00000202198**	RF00100	-1.592322867	Novel transcript
**ENSG00000243742**	RPLP0P2	-1.854651839	ribosomal protein lateral stalk subunit P0 pseudogene 2
**ENSG00000079102**	RUNX1T1	-2.50721877	RUNX1 translocation partner 1
**ENSG00000134548**	SPX	-1.699360273	spexin hormone
**ENSG00000110719**	TCIRG1	-2.871301647	T cell immune regulator 1, ATPase H+ transporting V0 subunit a3
**ENSG0000066056**	TIE1	-1.757477046	tyrosine kinase with immunoglobulin like and EGF like domains 1
**ENSG00000125355**	TMEM255A	-3.033187994	transmembrane protein 255A
**ENSG00000125657**	TNFSF9	-1.591705506	TNF superfamily member 9
**ENSG00000170396**	ZNF804A	-1.67965965	zinc finger protein 804A
