# Supplementary material for: Overexpression and Activation of αvβ3 Integrin Differentially Affects TGFβ2 Signaling in Human Trabecular Meshwork Cells
Source: Cells. 2021 Jul 29;10(8):1923. doi: 10.3390/cells10081923 (PMC8394542; doi:10.3390/cells10081923)
Supplement: Supplementary file 1 [file cells-10-01923-s001.zip › Table S5.pdf]

Table S5. Commonly upregulated genes and transcripts in cells overexpressing either a wild type (WTβ3) or a constitutively active (CAβ3) αvβ3 integrin.

Log_2 fold change (FC) values were reported in DGE analysis using EdgeR, and genes and transcripts that demonstrated a $Log_2 \geq 1.5$ FC were identified using R statistical computing and graphics software. Genes and transcripts are listed alphabetically.

**Ensembl ID**	**Gene Symbol**	**Log_2 FC WTβ3**	**Log_2 FC CAβ3**	**Description**
**ENSG00000175899**	A2M	2.546851196	2.190721295	alpha-2-macroglobulin
**ENSG00000270823**	AC007938.2	1.721227681	2.333318666	novel transcript, antisense to MEST
**ENSG00000249839**	AC011330.1	3.371762947	2.558261417	histidine acid phosphatase domain containing 2A pseudogene
**ENSG00000259753**	AC068234.1	9.595384645	6.518827194	novel transcript
**ENSG00000254673**	AC110275.1	1.569670313	1.553657788	novel transcript
**ENSG00000164742**	ADCY1	2.155402951	1.98806475	adenylate cyclase 1
**ENSG00000169083**	AR	1.743956491	2.023372026	androgen receptor
**ENSG00000158321**	AUTS2	2.072386152	3.522554032	AUTS2, activator of transcription and developmental regulator
**ENSG00000188848**	BEND4	3.026227118	4.745703048	BEN domain containing 4
**ENSG00000152785**	BMP3	1.523561956	3.820178962	bone morphogenetic protein 3
**ENSG00000224389**	C4B	1.511639907	2.296971491	complement C4B (Chido blood group)
**ENSG00000117477**	CCDC181	2.265861017	3.025809089	coiled-coil domain containing 181
**ENSG00000128510**	CPA4	1.511030785	3.022971372	carboxypeptidase A4
**ENSG00000135144**	DTX1	2.22806022	3.024584843	deltex E3 ubiquitin ligase 1
**ENSG00000130054**	FAM155B	2.633270588	2.493155194	family with sequence similarity 155 member B
**ENSG00000054983**	GALC	2.075155065	3.096050797	galactosylceramidase
**ENSG00000110328**	GALNT18	2.358499405	2.514794853	polypeptide N-acetylgalactosaminyltransferas e 18
**ENSG00000007237**	GAS7	1.512978922	3.210195626	growth arrest specific 7
**ENSG00000124194**	GDAP1L1	1.549983559	2.648958028	ganglioside induced differentiation associated protein 1 like 1
**ENSG00000112964**	GHR	3.441519165	2.810438978	growth hormone receptor

**ENSG00000179855**	GIPC3	1.821006768	2.428827304	GIPC PDZ domain containing family member 3
**ENSG00000065325**	GLP2R	1.941295934	2.365404281	glucagon like peptide 2 receptor
**ENSG00000170412**	GPRC5C	1.837777211	2.558563065	G protein-coupled receptor class C group 5 member C
**ENSG00000183454**	GRIN2A	2.183723762	2.107439598	glutamate ionotropic receptor NMDA type subunit 2A
**ENSG00000164116**	GUCY1A1	1.521115577	3.361417459	guanylate cyclase 1 soluble subunit alpha 1
**ENSG00000130600**	H19	1.523468872	2.269401426	H19, imprinted maternally expressed transcript
**ENSG00000274618**	HIST1H4F	1.632699824	2.340301588	histone cluster 1 H4 family member f
**ENSG00000125430**	HS3ST3B1	1.776234593	2.107503126	heparan sulfate-glucosamine 3-sulfotransferase 3B1
**ENSG00000142149**	HUNK	1.720255649	2.998820911	hormonally up-regulated Neu-associated kinase
**ENSG00000259207**	ITGB3	6.688975307	3.887676455	integrin subunit beta 3
**ENSG00000055118**	KCNH2	1.753339719	2.014887719	potassium voltage-gated channel subfamily H member 2
**ENSG00000003096**	KLHL13	3.485927333	2.470729486	kelch like family member 13
**ENSG00000169783**	LINGO1	1.509940943	2.335313903	leucine rich repeat and Ig domain containing 1
**ENSG00000136944**	LMX1B	1.524723261	1.658412873	LIM homeobox transcription factor 1 beta
**ENSG00000129295**	LRRC6	1.990976674	2.066274564	leucine rich repeat containing 6
**ENSG00000126217**	MCF2L	1.749244740	2.43608757	MCF.2 cell line derived transforming sequence like
**ENSG00000130675**	MNX1	2.423211431	4.367116869	motor neuron and pancreas homeobox 1
**ENSG00000151322**	NPAS3	1.795904567	1.737236428	neuronal PAS domain protein 3
**ENSG00000124374**	PAIP2B	1.99877162	2.289621046	poly(A) binding protein interacting protein 2B
**ENSG00000120324**	PCDHB10	1.925999419	3.392317423	protocadherin beta 10
**ENSG00000120328**	PCDHB12	1.614402947	2.547812606	protocadherin beta 12
**ENSG00000187372**	PCDHB13	1.590649225	2.369901446	protocadherin beta 13
**ENSG00000272674**	PCDHB16	1.995728907	2.085085236	protocadherin beta 16
**ENSG00000113209**	PCDHB5	1.560931899	2.252456238	protocadherin beta 5
**ENSG00000102109**	PCSK1N	2.625166846	2.641351326	proprotein convertase subtilisin/kexin type 1 inhibitor
**ENSG00000114631**	PODXL2	2.096004428	3.69766938	podocalyxin like 2

**ENSG00000074211**	PPP2R2C	2.255839625	2.103603309	protein phosphatase 2 regulatory subunit Bgamma
**ENSG00000103485**	QPRT	3.087796637	3.408612035	quinolinate phosphoribosyltransferase
**ENSG00000091428**	RAPGEF4	1.721583517	2.255201136	Rap guanine nucleotide exchange factor 4
**ENSG00000165731**	RET	1.831877241	2.411426246	ret proto-oncogene
**ENSG00000177181**	RIMKLA	2.421494183	2.883132189	ribosomal modification protein rimK like family member A
**ENSG00000092096**	SLC22A17	1.601709055	1.95807434	solute carrier family 22 member 17
**ENSG00000080503**	SMARCA2	2.152768611	2.318285941	SWI/SNF related, matrix associated, actin dependent regulator of chromatin, subfamily a, member 2
**ENSG00000101955**	SRPX	2.630509847	2.525241645	sushi repeat containing protein X-linked
**ENSG00000196562**	SULF2	1.887350742	2.277392509	sulfatase 2
**ENSG00000170379**	TCAF2	1.755030163	1.827709726	TRPM8 channel associated factor 2
**ENSG00000006042**	TMEM98	1.718692092	2.292466039	transmembrane protein 98
**ENSG00000255690**	TRIL	4.268021204	3.746862554	TLR4 interactor with leucine rich repeats
**ENSG00000162738**	VANGL2	3.262101997	3.758007605	VANGL planar cell polarity protein 2
**ENSG00000132821**	VSTM2L	2.740009491	2.597112733	V-set and transmembrane domain containing 2 like
**ENSG00000126970**	ZC4H2	1.560779227	1.886731068	zinc finger C4H2-type containing
**ENSG00000152926**	ZNF117	1.905433721	2.342995159	zinc finger protein 117
**ENSG00000197928**	ZNF677	1.783347861	1.662160198	zinc finger protein 677
**ENSG00000198429**	ZNF69	2.417505808	2.893734882	zinc finger protein 69
**ENSG00000188227**	ZNF793	2.017296376	2.443964647	zinc finger protein 793
