# Supplementary material for: Overexpression and Activation of αvβ3 Integrin Differentially Affects TGFβ2 Signaling in Human Trabecular Meshwork Cells
Source: Cells. 2021 Jul 29;10(8):1923. doi: 10.3390/cells10081923 (PMC8394542; doi:10.3390/cells10081923)
Supplement: Supplementary file 1 [file cells-10-01923-s001.zip › Table S6.pdf]

Table S6. Commonly downregulated genes and transcripts in cells overexpressing either a wild type (WTβ3) or a constitutively active (CAβ3) αvβ3 integrin.

Log_2 fold change (FC) values were reported in DGE analysis using EdgeR, and genes and transcripts that demonstrated a $Log_2 \leq -1.5$ FC were identified using R statistical computing and graphics software. Genes and transcripts are listed alphabetically.

Ensembl ID	Gene Symbol	Log_2 FC WTβ3	Log_2 FC CAβ3	Description
**ENSG00000141338**	ABCA8	-5.095589317	-2.980579945	ATP binding cassette subfamily A member 8
**ENSG00000127220**	ABHD8	-1.734154909	-1.859311682	abhydrolase domain containing 8
**ENSG00000265118**	AC134669.1	-1.854424156	-3.046258591	novel protein
**ENSG00000088543**	C3orf18	-2.183946766	-2.522262488	solute carrier family 35 member F6
**ENSG00000136999**	CCN3	-1.687291937	-2.187420855	cellular communication network factor 3
**ENSG00000125726**	CD70	-2.130170234	-1.745079339	CD70 molecule
**ENSG00000128965**	CHAC1	-4.710473005	-3.647274759	ChaC glutathione specific gamma-glutamylcyclotransferase 1
**ENSG00000174600**	CMKLR1	-2.074193754	-2.226520469	chemerin chemokine-like receptor 1
**ENSG00000175197**	DDIT3	-2.903410373	-2.251622005	DNA damage inducible transcript 3
**ENSG00000126860**	EVI2A	-1.806536706	-3.217884993	ecotropic viral integration site 2A
**ENSG00000132185**	FCRLA	-1.697799115	-2.271069757	Fc receptor like A
**ENSG00000130513**	GDF15	-1.728136069	-2.688561077	growth differentiation factor 15
**ENSG00000180353**	HCLS1	-1.577666113	-1.900570542	hematopoietic cell-specific Lyn substrate 1
**ENSG00000163395**	IGFN1	-3.581253567	-5.593492525	immunoglobulin-like and fibronectin type III domain containing 1
**ENSG00000150782**	IL18	-1.695609618	-3.747248872	interleukin 18
**ENSG00000136826**	KLF4	-1.6581479	-2.586682718	Kruppel like factor 4
**ENSG00000260676**	LINC01541	-8.921016897	-7.387253708	long intergenic non-protein coding RNA 1541
**ENSG00000259518**	LINC01583	-1.67473765	-1.743581189	long intergenic non-protein coding RNA 1583

**ENSG00000175471**	MCTP1	-1.61723307	-2.038949477	multiple C2 and transmembrane domain containing 1
**ENSG00000117122**	MFAP2	-1.889266239	-4.017772303	microfibril associated protein 2
**ENSG00000196611**	MMP1	-3.585185871	-6.351660168	matrix metallopeptidase 1
**ENSG00000144821**	MYH15	-1.561878888	-2.632268215	myosin heavy chain 15
**ENSG00000091129**	NRCAM	-2.311441565	-2.47152090	neuronal cell adhesion molecule
**ENSG00000181195**	PENK	-4.956812575	-4.22864203	proenkephalin
**ENSG00000182621**	PLCB1	-4.854838303	-2.001679576	phospholipase C beta 1
**ENSG00000099256**	PRTFDC1	-1.739074747	-2.158157087	phosphoribosyl transferase domain containing 1
**ENSG00000263426**	RN7SL471P	-1.537698057	-1.586087934	RNA, 7SL, cytoplasmic 471, pseudogene
**ENSG00000128228**	SDF2L1	-1.901822192	-2.276289361	stromal cell derived factor 2 like 1
**ENSG00000112837**	TBX18	-2.580444836	-1.734454771	T-box 18
**ENSG00000100234**	TIMP3	-1.572887624	-2.021798676	TIMP metallopeptidase inhibitor 3
**ENSG00000041982**	TNC	-2.246990957	-3.589927301	tenascin C
**ENSG00000121236**	TRIM6	-4.277405775	-2.617367483	tripartite motif containing 6
**ENSG00000261040**	WFDC21P	-1.654458803	-1.906100495	WAP four-disulfide core domain 21, pseudogene
**ENSG00000164684**	ZNF704	-2.670403494	-1.627879127	zinc finger protein 704
