# Supplementary material for: Overexpression and Activation of αvβ3 Integrin Differentially Affects TGFβ2 Signaling in Human Trabecular Meshwork Cells
Source: Cells. 2021 Jul 29;10(8):1923. doi: 10.3390/cells10081923 (PMC8394542; doi:10.3390/cells10081923)
Supplement: Supplementary file 1 [file cells-10-01923-s001.zip › Table S7.pdf]

Table S7: Classification of Protein-Coding Transcripts Altered in WTβ3 and/ or CAβ3 cells.

Downregulated transcripts are in red and upregulated transcripts are in black. Those transcripts that were either downregulated or upregulated in both cell lines are in bold. KEGG, DAVID Bioinformatics Resources, the Matrisome database and UniProt were used to classify the transcripts. The Miscellaneous Bio-annotation includes proteins that currently have no classification.

**Bio-annotation**	**# of Genes**	**Genes in WTβ3 Cells**	**# of Genes**	**Genes in CAβ3 Cells**
**Enzymes (includes protein kinases and glycosyltransferases)**	30	**PLCB1; CHAC1; MMP1;** HERC5; GALNT14; TIE1; PTPRN; CYP1B1<br>**QPRT; HUNK; CPA4; DTX1; GALNT18; HS3ST3B1; GALC; SMARCA2; ADCY1; GUCY1A1; RIMKLA; RET; SULF2;** B3GALNT1; ENPP4; CA9; PTGIS; CPS1; ATP8A2; QPCT; GALNT14	69	**PLCB1; CHAC1; MMP1;** PTGES; TENT5B; EYA2; PLEK2; ARHGAP24; SGIP1; TNFAIP3; CHST7; CHST6; PTPRO; ST6GALNAC5; MME; FA2H; DUSP5; SIK1; PDF; PCSK1; NUDT4B; EYA2; SPOCD1; PGBD5<br>**QPRT; HUNK; SULF2; CPA4; DTX1; GALNT18; HS3ST3B1; GALC; SMARCA2; ADCY1; GUCY1A1; RIMKLA; RET;** TLL1; USP51; DIO2; ABCB1; OAS2; NUDT11; PDE3A; KATNAL2; KYNU; DHRS2; EGLN3; CA4; DDO; ACADS; GUCY1A2; FGFR2; HPD; KSR2; CMPK2; OAS1; DTX4; SULF1; PRSS12; PTP4A3; PDE5A; CHDH; PDE8B; FGFR3; HR; CFI; ABAT; MEST
**Membrane trafficking**	13	**PLCB1**; **HCLS1**; **CCN3**<br>**MCF2L**; **LINGO1**; **GIPC3**; **ITGB3**; **GAS7**; **RAPGEF4**; **A2M;** TBC1D8B; SH3GL2; ADGRB1	27	**PLCB1**; **HCLS1**; **CCN3**; SYTL3; RAB27B; PLEK2; SGIP1; NRP2; ARHGAP24; MSLN<br>**RAPGEF4**; **GIPC3**; **A2M**; **MCF2L**; **LINGO1**; **ITGB3**; **GAS7**; SYT13; SRGAP3; DENND2D; ARHGAP22; DENND2C; DOC2B; AGAP2; EXPH5; SYT1
**Transcription (includes transcription factors, nuclear receptors and**	19	**KLF4**; **DDIT3; TBX18; ZNF704**; EBF1; EGR2; RUNX1T1; NUPR1<br>**ZNF677**; **ZC4H2**; **LMX1B**; **NPAS3**; **AR; ZNF793; ZNF69**;	57	**KLF4**; **DDIT3; TBX18; ZNF704**; FOSL1; SP140; MAFF; KLF2; BNC1; RELB; RFX8; EYA2; SPOCD1; LPXN<br>**ZNF677; ZC4H2; LMX1B**; **NPAS3**; **ZNF793; ZNF69**; **ZNF117; AR; SMARCA2; MNX1**;

**transcription machinery)**		**ZNF117**; **SMARCA2; MNX1;** ZNF626		EBF1; ISL1; SOX5; HOXC9; STAT5A; LHX1; PAX6; ALX4; LHX2; EBF4; DMRTA1; HOXA13; ZIC5; DLX4; FOXS1; ZNF347; ZNF429; ZNF730; ZNF641; MYCL; TXNIP; MYOCD; ZNF467; ZNF391; ZNF667; RASL11A; PPARGC1A; USP51; RNASEL; EID3; RGMA; HR; ATF7;
**Peptidases and inhibitors**	11	**ABHD8; MMP1; TIMP3**<br>**A2M**; **C4B**; **CPA4**; **PCSK1N**; CPS1; QPCT; ADGRB1; SERPINB9	19	**ABHD8; MMP1; TIMP3**; TNFAIP3; MME; SERPINE1; BIRC3; PCSK1<br>**A2M; C4B**; **CPA4**; **PCSK1N;** TLL1; USP51; C4A; PRSS12; ADGRL3; CFI; CPAMD8
**Exosome**	10	TCIRG1; ACTG2<br>**ITGB3**; **C4B**; **A2M;** SLC44A4; PLP1; TUBA4A; TC2N; CETP	19	PARVB; MBP; MME; RAB27B; SERPINE1; ANXA3; LPXN<br>**ITGB3**; **C4B**; **A2M**; ABCB1; MARVELD2; CA4; C4A; HPD; EFNB1; IFITM1; CFI; CRYAB
**Cell adhesion molecules**	14	**NRCAM;** CDH11; PCDH20; JAM2<br>**PCDHB13**; **PCDHB16**; **ITGB3**; **PCDHB5**; **PCDHB12**; **PODXL2 PCDHB10;** PCDHGB3; PCDHGB1; TENM4	28	**NRCAM;** MSLN; CNTN3; VCAM1; PCDHGA6<br>**PCDHB13**; **PCDHB16**; **ITGB3**; **PCDHB5**; **PCDHB12**; **PODXL2; PCDHB10;** DCHS1; PCDH1; PCDHB11; IGDCC4; PCDH18; CDH6; PCDHB14; NCAM2; SNED1; ADGRL3; EFNB1; TRO; CD33; MARVELD2; FLRT3; LRFN5;
**Cytoskeleton associated proteins**	10	**HCLS1; MYH15**; ACTG2; MAP7 KIAA1211L (CRACD)<br>**GAS7**; **LRRC6;** NHS; TUBA4A; ADAP2	16	**HCLS1; MYH15**; PARVB; VILL; MYBPH; LPXN<br>**GAS7**; **LRRC6**; ANK3; TMSB15A; KATNAL2; INA; IQGAP2; SORBS1; ABLIM1; RFLNA
**Extracellular Matrix Associated Proteins**[1]	15	**MFAP2; MMP1; TNC; TIMP3** COL11A1; TIE1<br>**VSTM2L**; **SULF2; A2M; SRPX**; POSTN; EMILIN1; COL8A2; SERPINB9; SEMA5B;	29	**MFAP2; MMP1; TNC; TIMP3;** VEGFA; BMPER; SERPINE1; WNT7B; TNFAIP6; COL13A1; GREM1<br>**VSTM2L**; **SULF2**; **A2M; SRPX**; SEMA6D; MFAP4; FREM2; KAZALD1; SEMA6A;

				SEMA3D; TLL1; COL14A1; VWA5A; WNT8B; SULF1; ANGPT1; EMILIN3; SPOCK1
**G-protein coupled receptors**	6	**CMKLR1;** OPRD1; GPR1 **GPRC5C**; **GLP2R;** ADGRB1;	10	**CMKLR1;** HTR7 **GPRC5C**; **GLP2R**; SSTR2; S1PR5; ADRA2C; ADGRL3; ACKR1; GPR156;
**Chromosome and associated proteins**	6	**AUTS2**; **PPP2R2C**; **SMARCA2; H4C6 (HIST1H4F)**; TUBA4A; SPAG4	14	H2BC6 (HIST1H2BE); PGBD5 **AUTS2**; **PPP2R2C**; **SMARCA2; H4C6 (HIST1H4F)**; TSPYL5; USP51; SYCE2; DCDC2; PPP2R2B; HR; USH1G; H1-1(HIST1H1A)
**Cytokine Interactions**	7	**GDF15; IL18; CD70**; TNFSF9; GDNF **TRIL; BMP3**	22	**GDF15; IL18; CD70**; CSF3; INHBB; VEGFA; IL1B; IL11; LIF; CXCL1; CXCL2; CXCL3; CXCL5; CXCL8; TNFAIP3 **TRIL; BMP3**; ANGPT1; BMP4; TGFB2; EFNB1; TRAF3IP3
**CD molecules**	6	**CD70**; **EVI2A;** JAM2; IL21R **ITGB3;** CDCP1	17	**CD70**; **EVI2A;** KLRC2; MME; CD274; CDCP1; EVI2B; VCAM1 **ITGB3**; CD33; ABCB1; BST2; FGFR2; FGFR3; IFITM1; ACKR1; NGFR
**Transporters**	8	**ABCA8; C3orf18 (SLC35F6); PENK** **SLC22A17**; CETP; SLC7A8; SLC44A4; RBP7	17	**ABCA8; C3orf18 (SLC35F6); PENK;** MFSD2A **SLC22A17**; CNNM1; ABCB1; SLC7A14; TMEM63C; NKAIN1; SLCO5A1; SLC8A2; SLC29A3; IFITM1; ATCAY; SLC19A3; REEP1
**Ubiquitin system**	4	**TRIM6**; HERC5 **DTX1**; **KLHL13**	11	**TRIM6**; BIRC3; KCTD14; TNFAIP3; TRIML2 **DTX1; KLHL13;** USP51; DTX4; KBTBD11; ARRDC4
**Protein phosphatases and associated proteins**	3	PCDH20; PTPRN **PPP2R2C**	11	PTPRO; SPOCD1; PPP4R4; DUSP5; EYA2; SYTL3 **PPP2R2C;** SLC7A14; PCDH1; PTP4A3; PPP2R2B
**Ion channels**	5	KCNS3	15	**GRIN2A**; **KCNH2**; **TCAF2**; **FAM155B**; ANO1; ANO4; CACNA2D2; P2RX7; KCNT2;

		**GRIN2A**; **KCNH2; TCAF2**; **FAM155B**		KCNJ8; CHRNB2; SCN9A; CHRNA3; ITPR2; TSPAN13
**Glycosaminoglycan binding proteins**	2	**TNC**; COL11A1	13	**TNC**; IL1B; VEGFA; CXCL8; WNT7B; COL13A1; SERPINE1; TNFAIP6<br>SLC8A2; FGFR2; BMP4; TGFB2; SLIT3
**mRNA biogenesis**	2	**PPP2R2C;** TUBA4A	5	TENT5B; GSPT2; MBP<br>**PPP2R2C**; PPP2R2B
**GTP-binding proteins**	7	RAB36; PLEKHG4<br>**RAPGEF4; MCF2L;** DIRAS3; RND2; ADAP2	9	RHOD; RAB27B; ARHGAP24 (FILGAP)<br>**RAPGEF4; MCF2L;** IQGAP2; RUNDC3A; SRGAP3; ARHGAP22
**Cytochrome p450**	2	CYP1B1; PTGIS	0	
**Mitochondrial biogenesis**	2	**GDAP1L1;** CHCHD10	2	**GDAP1L1**; PPARGC1A
**Cytokine receptors**	3	**CMKLR1;** IL21R<br>**GHR**	5	**CMKLR1;** IL31RA<br>**GHR;** ACKR1; NGFR
**Chaperones and folding catalysts**	0		4	DNAJB9; PCSK1<br>DNAJC12; CRYAB
**Spliceosome**	0		3	RNVU1-7<br>NOVA2; SRSF8
**DNA repair and recombination proteins**	0		2	PPP4R4<br>LRATD2 (FAM84B)
**Ribosome biogenesis**	1	ATP8A2	0	
**Translation factors**	0		1	GSPT2
**Miscellaneous**	26	**IGFN1**; **MCTP1; PRTFDC1; SDF2L1**; **FCRLA**; **HCLS1;** CRYBG1; ZNF804A; TMEM255A; CHCHD10; LRRC17; SPX; NIPSNAP1; MID1IP1	68	**IGFN1**; **MCTP1; PRTFDC1; SDF2L1**; **FCRLA**; **HCLS1;** LURAP1L; FAM167A; NUP50-DT; TMEM158; ARMCX2; TM4SF19; SBSN; ANKRD29; SH2D5; HPCAL1; TEX15; NPY; DTNA; SCG2; PRUNE2; EMP1; PAQR5; KDELC2; FCRLB; GCSAM; DKK1; S100A6; DNER; PHLDA2

	**CCDC181; H3BM21 (AC068234.1); TMEM98; BEND4; PAIP2B; VANGL2;** PLAC8; CARD11; NMU	**CCDC181; H3BM21 (AC068234.1); TMEM98; BEND4; PAIP2B**; **VANGL2;** FAM49A; LYPD6B; FAM13C; FAM225A; ZNF608; ARMH4; TMEM130; AMOT; SAMD11; ENDOD1; NPR3; ZNF66; CASC10; CASC15; LAMP5; SELENOP; TSPAN11; DMKN; APOC1; OLFM1; SNCAIP; SLITRK6; DLL1; C4BPA; SMAD6; MAGEH1; PKIB; LIN7A; TOX; SAMD5; HHIPL2; EFHD1
