# Supplementary material for: Overexpression and Activation of αvβ3 Integrin Differentially Affects TGFβ2 Signaling in Human Trabecular Meshwork Cells
Source: Cells. 2021 Jul 29;10(8):1923. doi: 10.3390/cells10081923 (PMC8394542; doi:10.3390/cells10081923)
Supplement: Supplementary file 1 [file cells-10-01923-s001.zip › Table S8.pdf]

Table S8: Classification of Non-coding Genes and Novel Transcripts exhibiting DGE in WTβ3 and/ or CAβ3 cells.

Downregulated transcripts are in red and upregulated transcripts are in black. Those transcripts that were either downregulated or upregulated in both cell lines are in bold. These transcripts were classified using the GeneCards database (https://www.genecards.org/). A total of 62 transcript types were detected in both cell lines.

Bio-annotation	# of genes	Genes WTβ3 Cells	# of genes	Genes CAβ3 Cells
**snRNA, snoRNA**	1	AL591806.3 (SNODB960)	3	RNVU1-7; SNORD3B-2; SNORD3B-1
**Open reading**	1	C3orf70	1	C2orf72
**Anti-sense**	4	CYP1B1-AS1<br>AC007938.2; SIRPG-AS1; DARS-AS1	10	AC004264.1; AC010247.1; SH3RF3-AS1; AC125611.3<br>AC007938.3; C4A-AS1; C4B-AS1; AC244517.11; HLA-F-AS1; PLCE1-AS1
**lincRNA, lncRNA**	6	**LINC01541**; **LINC01583**<br>AC008556.1; FP236383.2; FP236383.3; FP671120.4	9	**LINC01541; LINCO1583;** LINC00968; LINC02657<br>LINC02593; AC083967.1; AC005696.4; AC046168.2; AC008669.1
**pseudogene**	7	**WFDC21P; RN7SL471P;** RPLP0P2; AC113935.1<br>**AC011330.1 (HISPPD2A);** RPL13AP20; FER1L4	12	**WFDC21P; RN7SL471P;** RN7SL3; AC245297.1; NAMPTP1<br>**AC011330.1 (HISPPD2A);** AC099548.2; DUXAP8; DUXAP9; AL589743.1 (DUXAP10); HIST2H2BB; CASP17P
**microRNA**	1	**MIR675 (H19)**	2	MIR3142HG<br>**MIR675 (H19)**
**Ribosomal RNA**	4	RNA5-8SN1; RNA5-8SN2; RNA5-8SN3; RF00002	4	RNA5-8SN1; RNA5-8SN2; RNA5-8SN3; RF00002
**Novel Transcript**	3	**AC134669.1;** AC012181.1<br>**AC110275.1**	5	**AC134669.1;** AC048338.1; AL353150.1<br>**AC110275.1;** AC130304.1
