# Supplementary material for: Overexpression and Activation of αvβ3 Integrin Differentially Affects TGFβ2 Signaling in Human Trabecular Meshwork Cells
Source: Cells. 2021 Jul 29;10(8):1923. doi: 10.3390/cells10081923 (PMC8394542; doi:10.3390/cells10081923)
Supplement: Supplementary file 1 [file cells-10-01923-s001.zip › Table S9.pdf]

Table S9: Genes differentially expressed in CAβ3 cells that are targeted by TGFβ2, BMP and/or Wnt signaling.

Genes differentially expressed in CAβ3 cells were entered into the EMT-Regulome database (http://www.medsysbio.org/EMTRegulome) to identify those that are targeted by signaling involving TGFβ2, BMP and/or Wnt pathways (no citations indicated). In those instances where the database did not indicate an association with any pathway(s), supplementary literature searches were performed. The Log_2 FC relative to WTβ3 cells is indicated for each gene.

Functional Classification	Gene Name	Protein Name	CAβ3-EV Log_2 FC	Role in TGFβ, BMP or Wnt signaling
**Transcription**				
	**FOSL1**	FOS like 1, AP-1 transcription factor subunit	-1.98	Targeted by TGFβ [118]; Wnt [119]
	ISL1	ISL LIM Homeobox 1	8.81	Targeted by TGFβ; BMP [120]; Wnt [121]
	MYOCD	myocardin	2.94	Targeted by TGFβ
	**PAX6**	paired box 6	2.78	Targeted by TGFβ [122]; BMP [123]; Wnt [124]
	SOX5	SRY-box 5	3.49	Targeted by TGFβ; BMP
	STAT5A	signal transducer and activator of transcription 5A	3.32	Targeted by TGFβ
	ZNF117	zinc finger protein 117	2.34	Targeted by TGFβ
	ZNF347	zinc finger protein 347	1.78	Targeted by TGFβ
	ZNF608	zinc finger protein 608	2.63	Targeted by TGFβ
	ZNF641	zinc finger protein 641	2.17	Targeted by TGFβ; BMP
**Adhesion**				
	BMPER	BMP binding endothelial regulator	-2.07	Targeted by TGFβ
	CDH6	K-cadherin	1.97	Targeted by TGFβ; BMP
	CNTN3	contactin 3	-3.17	Targeted by TGFβ
	DCHS1	dachsous cadherin-related 1	3.65	Targeted by TGFβ; BMP
	NCAM2	neuronal cell adhesion molecule 2	1.78	Targeted by TGFβ
	NRCAM	neuronal cell adhesion molecule	-2.47	Targeted by TGFβ; BMP

	PCDH1	protocadherin 1	2.47	Targeted by TGFβ
	RGMA	repulsive guidance molecule BMP co-receptor a	2.16	Targeted by TGFβ
	SEMA6A	semaphorin 6A	1.55	Targeted by TGFβ; BMP
	SEMA6D	semaphorin 6D	4.35	Targeted by TGFβ; BMP
	VCAM1	vascular cell adhesion molecule 1	-2.07	Targeted by TGFβ; BMP
**ECM**				
	MMP1	matrix metallopeptidase 1	-6.35	Targeted by TGFβ; BMP [125]; Wnt [126]
	SERPINE1	serpin family E member 1	-1.71	Targeted by TGFβ
	TLL1	tolloid like 1	4.19	Targeted by TGFβ
	TNC	tenascin C	-3.59	Targets TGFβ [115]
**Cytokines**				
	BMP3	bone morphogenetic protein 3	3.54	Targeted by TGFβ; BMP
	IL11	interleukin 11	-2.05	Targeted by TGFβ
	INHBB	inhibin subunit beta B	-1.65	Targeted by TGFβ; BMP4
	SMAD6	SMAD family member 6	2.19	Targeted by TGFβ
	TGFB2	transforming growth factor beta 2	1.63	Targeted by BMP [73]
	TNFAIP3	TNF alpha induced protein 3	-1.92	Targeted by TGFβ; BMP
	WNT7B	Wnt family member 7B	1.82	Targeted by TGFβ [127]; BMP [128]
	WNT8B	Wnt family member 8B	1.90	Targeted by TGFβ; BMP
**Cytoskeleton**				
	AGAP2	ArfGAP with GTPase domain, ankyrin repeat and PH domain 2	1.77	Targeted by TGFβ
	ARHGAP22	Rho GTPase activating protein 22	2.24	Targeted by TGFβ
	ARHGAP24	Rho GTPase activating protein 24	2.04	Targeted by BMP
	GAS7	growth arrest specific 7	3.21	Targeted by TGFβ
	INA	internexin neuronal intermediate filament protein alpha	1.93	Targeted by TGFβ
	IQGAP2	IQ motif containing GTPase activating protein 2	2.59	Targeted by TGFβ; Wnt [129]
	MCF2L	MCF.2 cell line derived transforming sequence like	2.44	Targeted by BMP

	MYH15	myosin heavy chain 15	-2.10	Targeted by TGFβ [130]
	NHS	NHS actin remodeling regulator	-1.02	Targeted by TGFβ; BMP
	RAPGEF4	Rap guanine nucleotide exchange factor 4	2.26	Targeted by TGFβ
	RUNDC3A	RUN domain containing 3A	1.68	Targeted by TGFβ
	SORBS1	sorbin and SH3 domain containing 1	2.15	Targeted by TGFβ; BMP
	SRGAP3	SLIT-ROBO Rho GTPase activating protein 3	2.81	Targeted by BMP
**Miscellaneous**				
	TXNIP	thioredoxin interacting protein	3.64	Targeted by TGFβ; BMP
	MBP	myelin basic protein	-2.62	Targeted by TGFβ [131]
